# In Vivo Biocompatibility Study on Functional Nanostructures Containing Bioactive Glass and Plant Extracts for Implantology

**DOI:** 10.3390/ijms25084249

**Published:** 2024-04-11

**Authors:** Laura Floroian, Mihaela Badea

**Affiliations:** 1 Faculty of Electrical Engineering and Computer Science, Transilvania University of Brasov, Romania, No. 1, Politehnicii St., 500031 Brașov, Romania; 2 Faculty of Medicine, Transilvania University of Brasov, Romania, No. 56, Nicolae Bălcescu St., 500019 Brașov, Romania; mihaela.badea@unitbv.ro

**Keywords:** in vivo study, plant extract, orthopedic implant

## Abstract

In this paper, the in vivo behavior of orthopedic implants covered with thin films obtained by matrix-assisted pulsed laser evaporation and containing bioactive glass, a polymer, and natural plant extract was evaluated. In vivo testing was performed by carrying out a study on guinea pigs who had coated metallic screws inserted in them and also controls, following the regulations of European laws regarding the use of animals in scientific studies. After 26 weeks from implantation, the guinea pigs were subjected to X-ray analyses to observe the evolution of osteointegration over time; the guinea pigs’ blood was collected for the detection of enzymatic activity and to measure values for urea, creatinine, blood glucose, alkaline phosphatase, pancreatic amylase, total protein, and glutamate pyruvate transaminase to see the extent to which the body was affected by the introduction of the implant. Moreover, a histopathological assessment of the following vital organs was carried out: heart, brain, liver, and spleen. We also assessed implanted bone with adjacent tissue. Our studies did not find significant variations in biochemical and histological results compared to the control group or significant adverse effects caused by the implant coating in terms of tissue compatibility, inflammatory reactions, and systemic effects.

## 1. Introduction

Orthopedic implants play a crucial role in the field of medical science by providing support and restoring functionality to damaged or diseased bones and joints. To improve the success and longevity of these implants, researchers have been exploring the use of thin films from various materials or combinations of materials as coatings on implant surfaces [1,2,3,4,5,6]. Thin films offer a range of benefits, including enhanced biocompatibility [7], improved osseointegration [8], reduced infection rates [9], and controlled drug release [10]. Most modern approaches in this field involve the use of films containing plant extracts for implant covering; this is an area of research that explores the potential benefits of incorporating natural compounds into thin-film coatings [11,12,13,14]. Plant extracts contain a wide range of bioactive compounds, such as polyphenols, flavonoids, terpenoids, and alkaloids, which have shown various beneficial properties, including antioxidant, antimicrobial, anti-inflammatory, and osteogenic activities. These compounds have the potential to enhance the performance of orthopedic implants when incorporated into thin-film coatings.

Researchers have been exploring various plant extracts and their incorporation into thin-film coatings for orthopedic implants. Commonly used plant extracts include green tea extract [15], curcumin (from turmeric) [16], aloe vera [17], chamomile, and ginkgo biloba, among others. These extracts have shown promising results in enhancing biocompatibility, antimicrobial activity, and tissue regeneration in preclinical studies.

The fabrication techniques for incorporating plant extracts into thin films can vary, including methods such as electrospinning, layer-by-layer assembly, and physical vapor deposition. The choice of technique depends on the specific properties of the plant extract, the desired film characteristics, and the intended application.

While the use of plant extract-containing films for implant covering is still an active area of research, it shows potential for improving the performance of orthopedic implants by harnessing the natural properties of these plant-derived compounds.

Our team has proposed and implemented a thin double-layered structure fabricated by matrix-assisted pulsed laser evaporation (MAPLE) to cover a metallic implant [18]. The synthesized nanostructure consists of two overlapping layers: the lower layer comprises a biocompatible polymer for anticorrosive protection, and the upper one consists of bioactive glass incorporating antimicrobial plant extract, acting as a drug delivery system. Morphology, composition, adherence, ability for drug delivery, and biological properties (cytotoxicity and antimicrobial effect) were studied and validated by in vitro tests. The structures proved compact and stable, conserving a remarkable drug delivery ability for more than 21 days, i.e., enough to ensure long-term microbe eradication.

The bioactive compounds from plant extracts promote cell adhesion, proliferation, and differentiation, thereby enhancing tissue integration and reducing the risk of implant rejection. Plant extracts possess natural antimicrobial properties, making them effective in preventing bacterial colonization and reducing the risk of implant-associated infections. By incorporating plant extracts with antimicrobial activity into thin-film coatings, the films can create a protective barrier against bacteria, enhancing the long-term success of orthopedic implants.

Inflammation is a common response after implantation, which can hinder proper healing and the integration of the implant. Certain plant extracts have demonstrated anti-inflammatory properties by modulating inflammatory pathways and reducing inflammatory markers [19,20]. Thin-film coatings containing these extracts can help minimize inflammation at the implant site and promote better healing. Also, plant extracts have been found to stimulate bone formation and enhance osteogenic activity. When incorporated into thin-film coatings, these extracts can promote osseointegration, leading to improvements in the stability and longevity of orthopedic implants [21,22,23]. This work describes the final validation of a structure deposited over implants by in vivo tests, which are crucial. The study was conducted according to the international standards [24].

These types of tests involve the evaluation of implant performance and biocompatibility in living organisms, typically animal models. First, in vivo tests provide valuable information about the biocompatibility of thin-film coatings. They help determine the tissue response, inflammatory reactions, and the body’s acceptance of the implant with the coating. These tests are essential to ensure that the coating does not cause adverse reactions such as inflammation, infection, or immune responses. At the same time, in vivo studies assess the integration of the implant with the surrounding tissues [25]. They help to determine the degree of osseointegration, which is the direct structural and functional connection between the implant and the bone. In vivo tests provide insights into the strength and stability of the implant-coating system and its long-term performance. Moreover, in vivo testing allows for the evaluation of the functional performance of the implant with thin-film coating. This can include assessing the range of motion, load-bearing capacity, and biomechanical properties. Such evaluations provide valuable data on the ability of the coating to support normal physiological function.

In our work, some blood was used immediately after harvesting to quantify urea, creatinine, glycemia, alkaline phosphatase, pancreatic amylase, total serum protein, and glutamate pyruvate transaminase (TGP) to analyze how the body was affected by implant insertion. Also, enzyme activity was evaluated by measuring superoxide dismutase, catalase, and glutathione peroxidase.

Urea is the main nitrogenous final product of the metabolism of amino acids [26], originating from the splitting of proteins in the stomach and intestine under the action of proteolytic enzymes and their absorption through the intestinal wall [27]. The main seat of urea formation is the liver, but growing tissue (for example, embryonic or tumor tissue) also has the ability to form urea from arginine. Most of the urea is eliminated by glomerular filtration; 40–60% is redistributed in the blood depending on the tubular flow and the antidiuretic hormone.

Urea in blood and urine varies in direct proportion to the protein diet and is inversely proportional to cellular anabolism during growth, pregnancy, and convalescence [28]. Serum urea is primarily used as a measure of renal or metabolic dysfunction, but it also provides important information about liver function and dietary protein intake [29].

Creatinine is creatine anhydride (methylguanidylacetic acid) and represents its elimination form; it is formed in the muscle tissue. Creatine is synthesized in the liver, and after its release, it is taken up by the muscles in a percentage of 98.6%, where phosphorylation takes place, with this form having an important role in storing muscle energy [30]. When this muscle energy is required for the needs of metabolic processes, phosphocreatine is split to creatinine. The amount of creatine converted into creatinine is maintained at a constant level, which is directly related to the body’s muscle tissue mass.

The main utility of serum creatinine determination is the diagnosis of renal failure [31]. The disturbance of renal function reduces creatinine excretion, causing an increase in serum creatinine. Thus, creatinine concentrations provide an approximation of the glomerular filtration rate.

Amylase is an enzyme produced in the pancreas and salivary glands that hydrolyzes starch, glycogen, and related polysaccharides to simple sugars. Amylase is also secreted by the lining of the small intestine, ovaries, placenta, and fallopian tubes [32]. Pancreatic amylase is more thermally labile than salivary amylase, has tissue specificity, and is secreted by acinar cells. The dosage of pancreatic amylase gives indications about how the pancreas works, with the values obtained being important for establishing a diagnosis. Pancreatic amylase is generally the screening test for the diagnosis of acute pancreatitis, with its values increasing 4–6 times above the reference limit [33].

Proteins have a vital role in the normal functioning of the body; therefore, a test that determines total serum proteins is usually included in the list of investigations carried out periodically as a screening method. More than 300 proteins are found in plasma. The test that analyzes the level of total proteins does not count them but determines whether we have enough for our body to function normally. A protein level that is too high or too low can lead to weight loss, fatigue, and inflammatory diseases [34]. The determination of total serum proteins can help diagnose liver and kidney diseases, among others. Proteins are found as a component in enzymes, enzyme inhibitors, clotting factors, antibodies, and transporter proteins. Proteins play the most important role in maintaining osmotic pressure. 

Establishing the level of total serum proteins [35] helps in the investigation of edematous syndromes, the diagnosis of conditions accompanied by hypercatabolism such as hyperthyroidism, inflammations, neoplasias, chronic diseases, screening for the detection of gammopathy.

Alkaline phosphatase is an enzyme that belongs to the class of hydrolases and is found in the liver, digestive system, kidneys, and bones. Bone alkaline phosphatase is produced in osteoblasts and plays a role in the formation of bone tissue [36]. The laboratory test measures the activity of enzymes in the body and helps detect liver or bone disorders.

Increased values for alkaline phosphatase indicate the presence of a condition but cannot show whether the liver or bile ducts are functioning problematically. For this reason, in most cases, alkaline phosphatase is determined together with other specific analyses. It can also be used in oncological diagnosis [37], especially for bone tumors, Paget’s disease, and liver cancers [38].

Low alkaline phosphatase, that is a value below the reference range for the patient’s age and sex, can be seen in those with familial bone disease (hypophosphatasia), achondroplasia, cretinism, malnutrition, vitamin C deficiency, zinc deficiency, and magnesium deficiency, as well as in women with osteoporosis who have entered menopause [39].

Glutamate pyruvate transaminase is an enzyme found mostly in the liver and, to a lesser extent, in the kidneys, myocardium, skeletal muscles, and pancreas, with a role in enzymatic reactions that use proteins as an energy source [40]. The absolute values of the enzyme do not correlate directly with the severity of the organ’s suffering, as serial, periodic determinations of the compound are necessary to monitor the evolution of the disease and establish a prognosis in patients with this condition. The administration of medication (acetaminophen) or substances with a liver-toxic effect (e.g., alcohol consumption) represent other situations in which an increase in TGP values is recorded.

Increases of up to 20–100 times compared to the normal value are specific to acute viral and toxic drug hepatitis, while multiplication by 2 or 3 of the physiological values occurs in hepatic steatosis (fatty liver). Liver metastases are accompanied by moderate increases in TGP, and in the case of a diagnosis of primary hepatoma, the changes are minimal [41].

Decreases in TGP values are found in urinary infections, neoplasia (hepatocellular carcinoma), and pyridoxal phosphate deficiency (found in malnutrition and alcoholic hepatitis [42].

Superoxide dismutase (SOD) is an enzyme that catalyzes the dismutation of superoxide (O_2_•) into hydrogen peroxide (H_2_O_2_) and molecular oxygen (O_2_). Superoxide is a reactive oxygen species that can be produced in excess in inflammatory and oxidative processes [43]. SOD helps to eliminate superoxide and prevent the formation of other more harmful reactive oxygen species. This can protect tissues against implant-induced oxidative stress and promote healing and regeneration processes. SOD is widely distributed in humans, in all other mammals, and in most chordates. It occurs in high concentrations in the liver, heart, brain, kidney, and erythrocytes. Some enzymes (such as NADPH oxidase) are used by human white blood cells to generate superoxide and other reactive oxygen species to kill bacteria. The amount of SOD present in cellular and extracellular environments is crucial for the prevention of diseases linked to oxidative stress [44]. 

Catalase (oxidoreductase, CAT) is an omnipresent antioxidant enzyme that exists in most aerobic cells. Catalase is involved in the detoxification of hydrogen peroxide (H_2_O_2_), a reactive oxygen species, which is a toxic product of both pathogenic reactive oxygen species production and normal aerobic metabolism. In human beings, the highest levels of CAT are in the kidney, liver, and erythrocytes, where it is believed to account for the majority of H_2_O_2_ decomposition [45]. The measurement of catalase activity can provide information about the ability of the antioxidant system to cope with the stress induced by orthopedic implants.

Glutathione peroxidase (GPx) is an intracellular antioxidant enzyme that reduces hydrogen peroxide to water and limits its harmful effects [46]. By limiting hydrogen peroxide accumulation, GPx also modulates processes that involve it, like mitochondrial function, growth factor-mediated signal transduction, and the maintenance of normal thiol redox balance. The regulation and removal of hydrogen peroxide prevents the formation of the highly reactive and damaging hydroxyl radical, which can be formed by the reaction of hydrogen peroxide with Fe^2+^ (Fenton reaction) and may protect against oxidative stress [47]. GPx-1 is an isoform of GPx, one of several cellular enzymes that may modulate overall redox stress. Decreased GPx-1 activity can promote susceptibility to oxidative stress by allowing for the accumulation of harmful oxidants, whereas excess GPx-1 may promote reductive stress, characterized by a lack of essential ROS needed for cellular signaling processes. Excess oxidants or the loss of essential ROS can lead to diminished cell growth and promote apoptotic pathways [48].

## 2. Results and Discussion

Because the processes in the guinea pig body are carried out much faster than in humans [49], to obtain information on the behavior of the implant and the human body for a long time after implantation, we analyzed the implant and the body of each guinea pig at just 26 weeks after implantation. At that moment, the guinea pigs were subjected to another surgical procedure to remove the implant from the body, and at the same time, blood and organ harvests were collected from all the animals studied. 

### 2.1. X-ray Imaging

**Figure 1:**
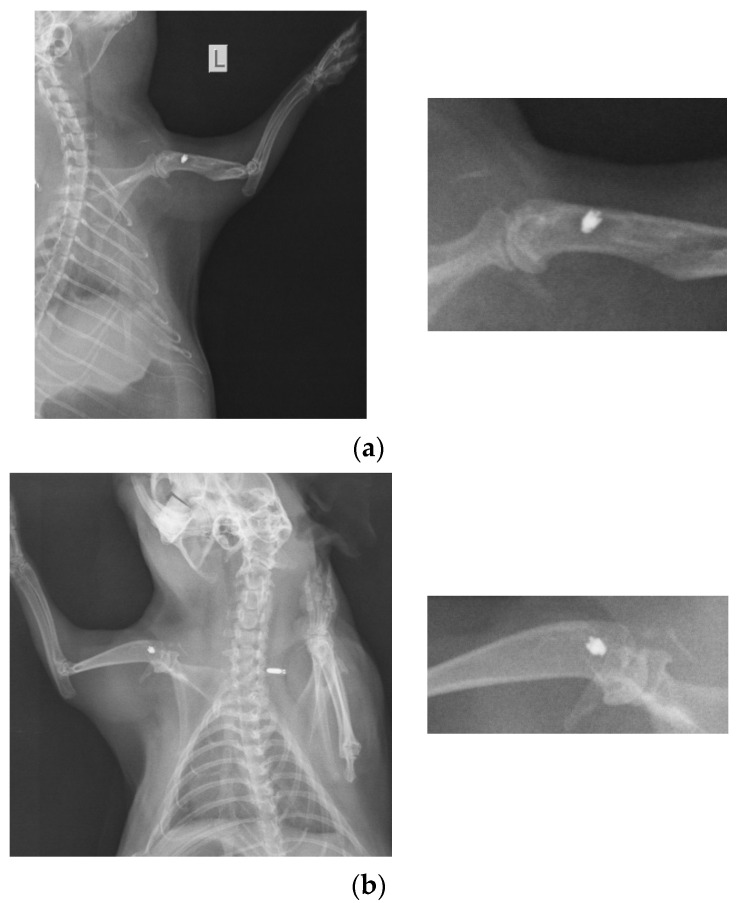

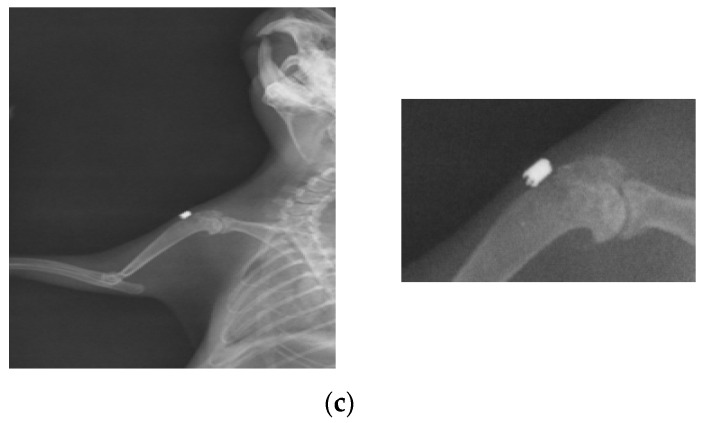
RX images of implants inserted in the humerus of guinea pigs: (**a**) lot I, (**b**) lot II, and (**c**) lot III.

At 26 weeks after implantation, the guinea pigs were again subjected to X-ray analyses to observe the evolution of osteointegration over time. Shown below are some radiographs and the significant details of them (Figure 1). There is good bone implant stability and good fixation (Figure 1a,b). In the case of a single guinea pig, the implant moves out of the bone and becomes parallel to the bone, as shown in Figure 1c. This could be due not necesarily an incorrect implantation but also to the fact that the animal feels a foreign body under the skin and does its best to remove it. For this reason, the implantations were performed carefully in areas where guinea pigs cannot reach with their teeth or claws.

### 2.2. EDS Analyses on Implants Removed from the Body 

The results of the quantitative compositional analysis of stainless steel (OL) implant coated with BG + neem/PMMA layer (referred to as BGN) are presented in Table 1. In addition to the components of hydroxyapatite (HA) (i.e., Ca, P, and O), EDS analyses revealed the presence of some trace elements (Na, Mg, F) characteristic of the bone mineral phase. In addition to the above-mentioned elements, which are naturally found in the chemical composition of healthy bone tissue, other species characteristic of the steel substrate could also be detected.

The Ca/P molar ratio of the material was ~1.30, indicating, according to FTIR results, the presence of carbonate apatite [50], which means that consistent osteointegration has taken place.

### 2.3. Blood Analyses

The harvested blood was used immediately after collection to measure values for urea, creatinine, blood glucose, alkaline phosphatase, pancreatic amylase, total protein, and TGP to see the extent to which the body was affected by the introduction of the implant of titanium (Ti), stainless steel, or stainless steel coated. The obtained values are presented in Table 2, also for guinea pigs without implant (WI). That table also contains normal blood analysis values for guinea pigs from the literature [51].

**Figure 2:**
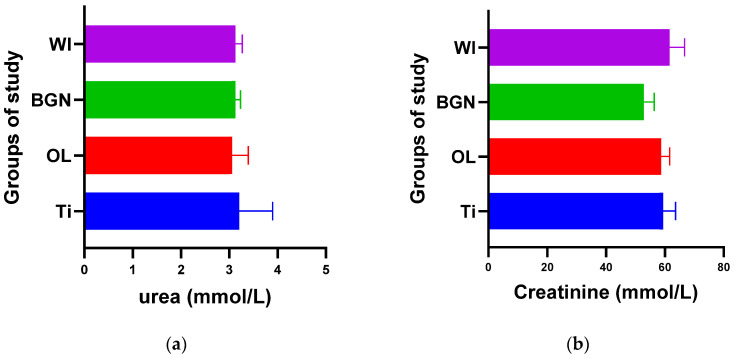
Urea (**a**) and creatinine (**b**) values of guinea pig groups.

**Figure 3:**
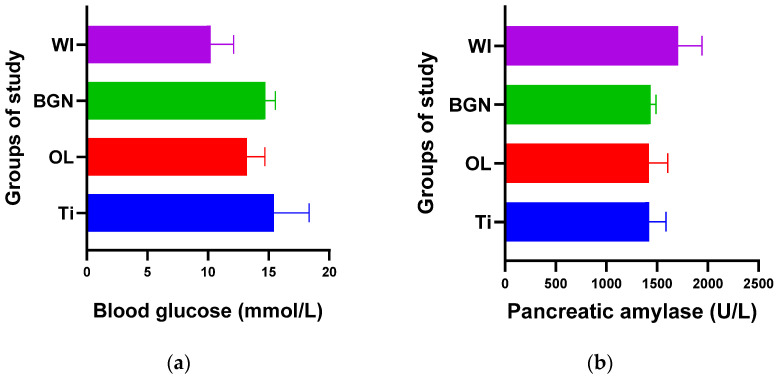
Blood glucose (**a**) and pancreatic amylase (**b**) values of guinea pig groups.

**Figure 4:**
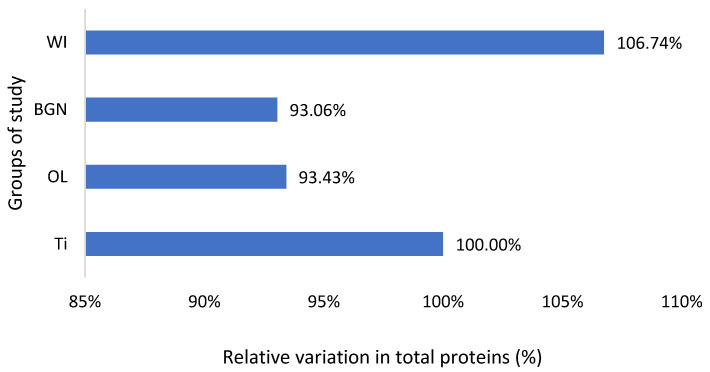
Relative variation in total proteins of guinea pig groups versus the group with the Ti implant.

**Figure 5:**
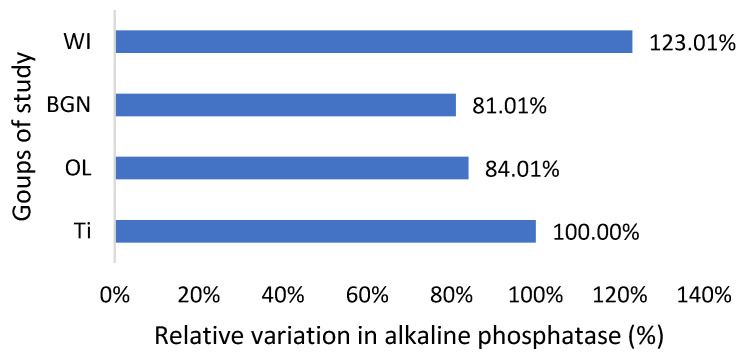
Relative variation in alkaline phosphatase of guinea pig groups versus the group with the Ti implant.

**Figure 6:**
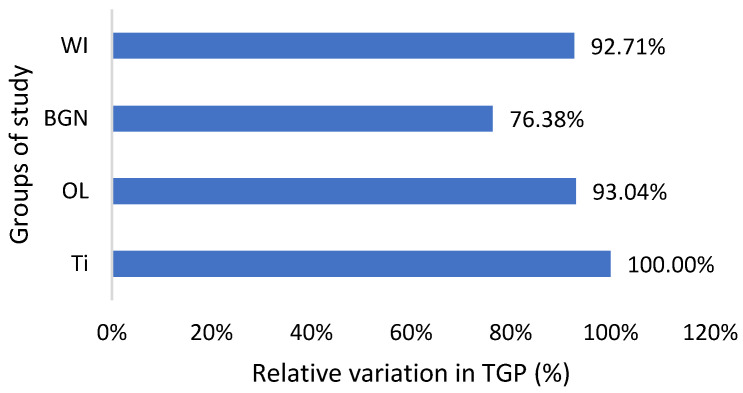
Relative variation in TGP of guinea pig groups versus the group with the Ti implant.

The graphs in Figure 2, Figure 3, Figure 4, Figure 5 and Figure 6 compare all the values with those measured for the titanium implant because the process for the validation of the in vivo tests requires reporting related to an implant made from a commonly used and accepted material, with established characteristics, which, in bone implantology, is titanium.

Figure 2a shows that the process of urea formation and elimination worked properly. Lower urea values compared to Ti values can suggest a more rapid formation of new bone tissue, as in vitro tests have demonstrated [18].

Figure 2b does not show an increase in serum creatinine in the case of implantation, instead showing a slight decrease, which proves the good functioning of the kidneys is not affected by the introduction or presence of the implant.

From the point of view of blood glucose determination, Figure 3a contains values obtained for all four groups of study, and we observed a slight increase in all cases with implants (Ti, OL, or BGN) compared to the cases without implants (WI), a normal reaction for a stressed body in which a foreign body is introduced.

Comparing the Ti and BGN groups, we observed smaller blood glucose values in the animals with implants coated with BGN because of its plant component, neem, which is recognized for such an effect [52].

In our experiment, measured values of pancreatic amylase were in the normal range in both the implanted and not-implanted guinea pigs, which underlined that the pancreas is not affected by the presence of the implant in the body. 

Also, the measured values of total protein are slightly lower than the normal ones, including those of animals without implants, but very close to each other (Figure 4). This fact, along with the results of the analyses described below, leads to the hypothesis of the existence of a lower-protein diet of the animals during the study. Proteins can adsorb on metal surfaces, and such protein adsorption layers can induce corrosion protection by blocking the active sites of the surface [53].

Regarding the alkaline phosphatase values obtained for the guinea pigs implanted with all types of implants, they are smaller than those for the pigs without implants (Figure 5). This could be related to the fact that bone tissue reconstruction is magnesium-consuming. Those with the BGN implant demonstrated a bigger rate of bone growth [18], as determined by in vitro testing, and the relative variation in alkaline phosphatase was bigger, which shows a good correlation.

Magnesium is a very important component of bone structure, an indispensable component for the absorption of calcium from food and its fixation at the bone level [54]. Magnesium stored in bones is not passive but contributes to bone stabilization, growth, and mineralization. During the bone synthesis process, intensified by the coating of the implant, the need for magnesium increases, and its consumption leads to increased absorption from the blood, hence the existing magnesium deficit.

As can be seen in Table 2, the TGP values are not in the normal range; instead, they are almost half of the minimum normal value for all study groups, which is bad news. Also, there are lower alkaline phosphatase and total protein values, all of which suggest that the nutrition of the guinea pigs was insufficient. In the studies that will follow, it will be necessary to change guinea pigs’ diet with a much more protein-rich one. The good news is that the TGP values measured for the guinea pigs with BGN or OL implants do not show an increase, but they are smaller than for the animals with the Ti implants (Figure 6). Low levels are generally considered good and are usually not a cause for concern. However, in some cases, a low TGP can be a result of an underlying medical condition, such as vitamin B6 deficiency. 

### 2.4. Detection of Other Enzymatic Activity

The introduction of an implant into the body naturally produces oxidative stress [55], a fact highlighted by the significant changes in enzyme activity for all three monitored enzymes.

**Figure 7:**
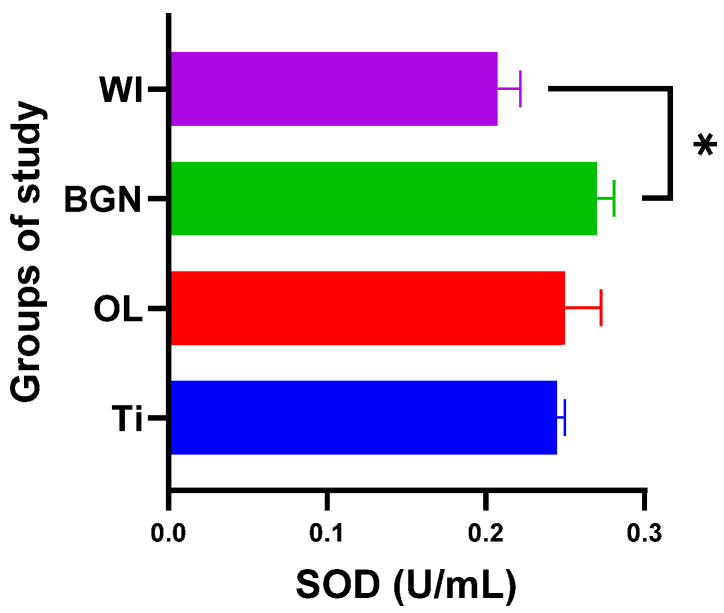
Superoxide dismutase values of guinea pig groups (* level of significance *p* < 0.5).

Analyzing the obtained results, we noticed that the presence of the BGN implant in the body causes it to release the largest amounts of SOD (see Figure 7), which can help to quickly eliminate superoxide and prevent the formation of other more harmful reactive oxygen species. This protects the tissues against the oxidative stress induced by the implant and promotes the healing and regeneration processes.

**Figure 8:**
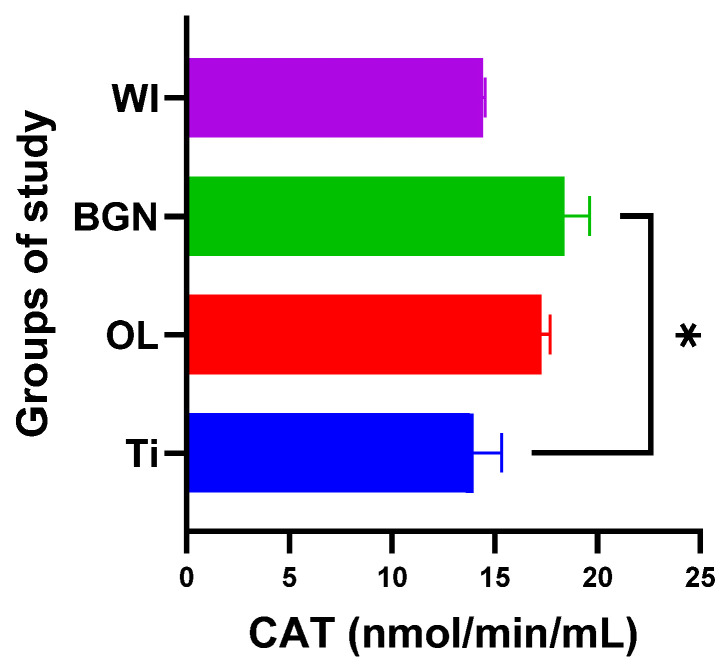
Catalase values of guinea pig groups (* level of significance *p* < 0.5).

Analyzing the values obtained for CAT, we noticed that the introduction of the titanium implant in the body leads to a slight decrease in catalase activity, and it seems that the antioxidant system has a weak capacity to deal with the oxidative stress induced by the implant. Things are completely different in the cases of the OL and BGN implants, where catalase has a pronounced activity, especially in the BGN group, the calculations for which generated statistically significant results (see Figure 8).

**Figure 9:**
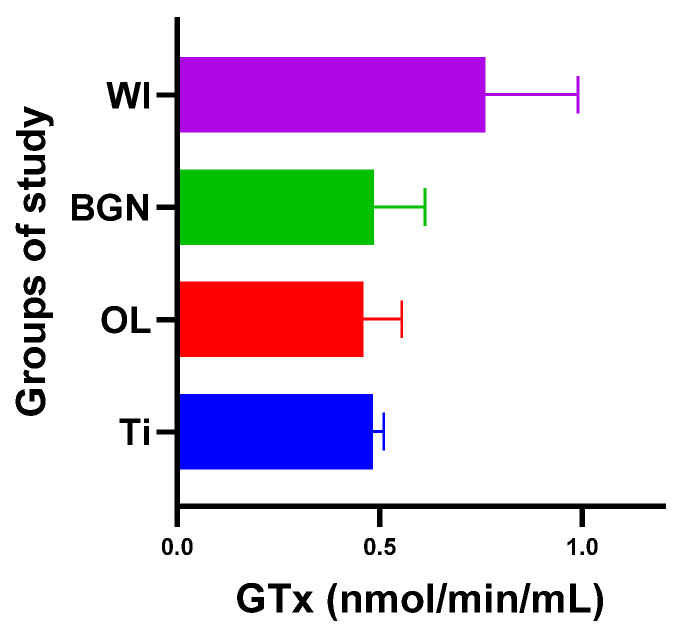
Glutathione peroxidase values of guinea pig groups.

Regarding GPx, the body reaction closest to the case of the guinea pigs with a titanium implant was obtained in the BGN group (Figure 9).

All this shows that the best response to the oxidative stress generated by the introduction of the implant into the body was recorded in guinea pigs with thin film-coated implants rather than those with Ti or stainless-steel implants.

The results obtained for the SOD, CAT, and GPx activities are summarized in Table 3.

Oxidative stress is a factor of the initiation of inflammation, fibrosis in various organs, genotoxicity, the inhibition of cell multiplication, and, finally, cell death, and obtaining lower values for oxidative stress in our work means there is a lower risk of these effects occurring when implanting screws coated with such nanocomposite thin films into the human body.

### 2.5. Histopathology Studies

**Figure 10:**
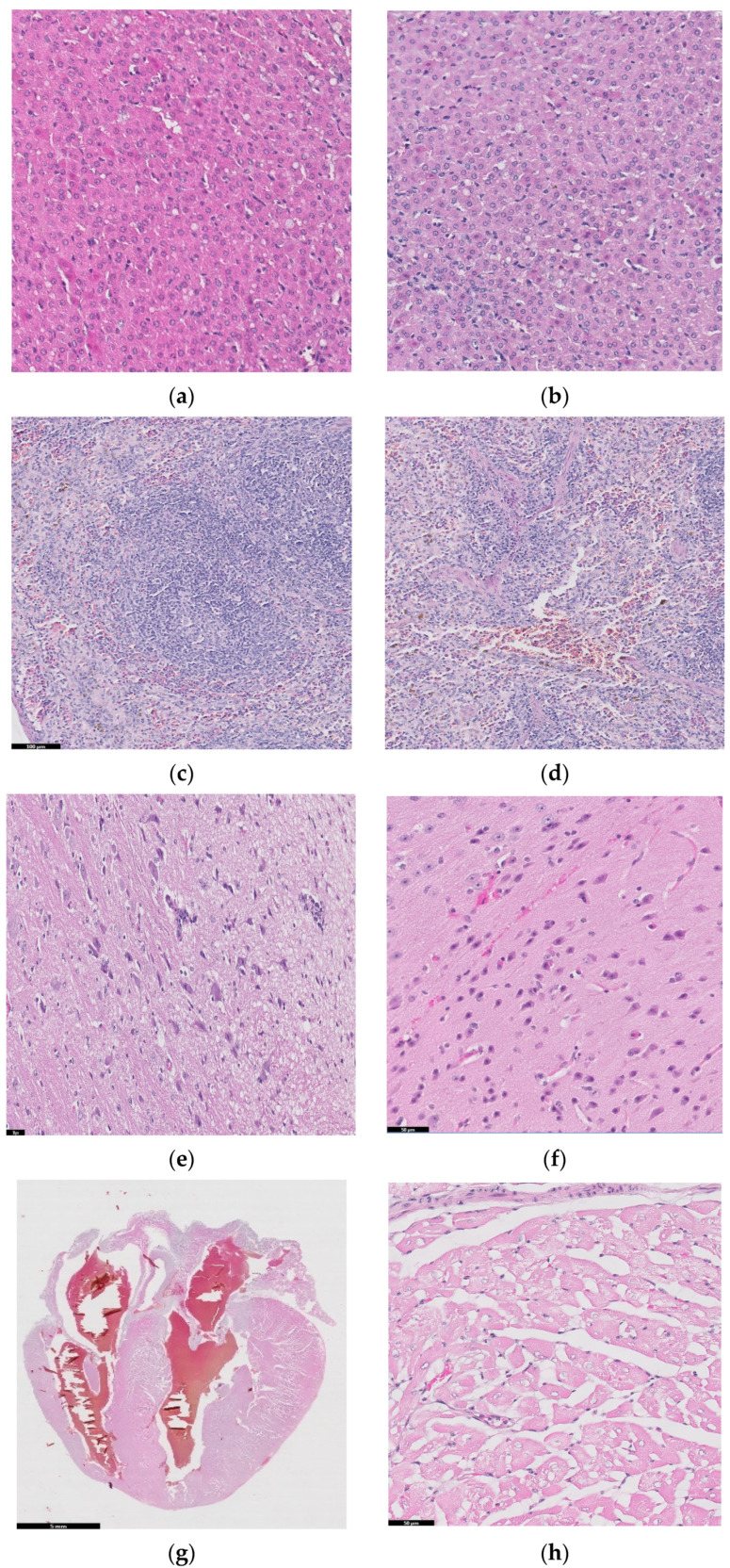

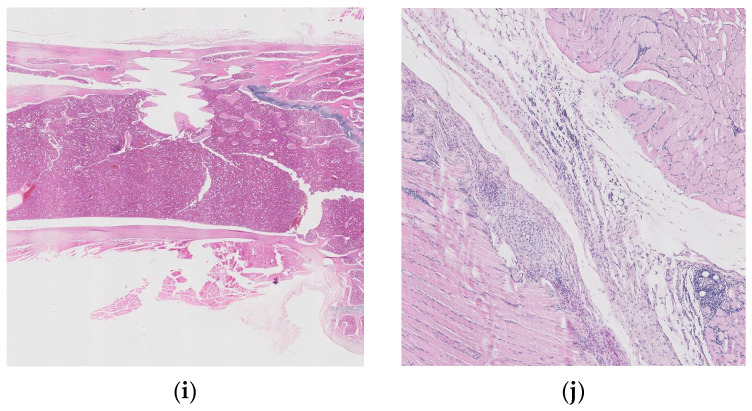
Histopathological images for liver (**a**,**b**), spleen (**c**,**d**), brain (**e**,**f**), heart (**g**,**h**), bone (**i**), and muscle (**j**).

Figure 10 shows histopathological images for guinea pigs’ organs after 26 weeks from implantation: liver (a, b), spleen (c, d), brain (e, f), heart (g, h), bone, (i) and muscle (j).

A liver sample of approximately 7 × 5 × 2.4 cm was sectioned through the left and right lateral lobes, processed, and embedded in paraffin. Figure 10a,b are diffuse; the hepatocytes show predominantly optically empty cytoplasm, rarely (periportal) with granular appearance, eosinophilic, central nucleus, rarely binucleated. The blood vessels and bile ducts do not show changes. Panlobular hepatic glycogenosis can be interpreted. Glycogenosis can be caused by increased glycogen synthesis (diets rich in carbohydrates, the administration of corticosteroids, etc.) or in glycogen storage diseases with systemic manifestation.

Spleen samples (2.9 × 1.6 × 0.5 cm) were sectioned in four slices parallel to the longitudinal axis, and two of which were processed and embedded in paraffin. Red pulp is well represented, with areas of extramedullary hematopoiesis and the accumulation of hemosiderin, and white pulp is represented by splenic follicles, occasionally with hyperplasia of the germinal centers. No pathological changes in the spleen were detected in the examined sections.

Two longitudinal sections, one paramedian and one sagittal through half of the hemisphere of brain, were processed and embedded in paraffin. In the examined sections, the nervous tissue did not show any pathological changes.

Cord of 2.5 × 2 × 1.6 cm with hemopericardium was examined. Parasagittal sectioning through the heart, including the four chambers and, partially, the great vessels, was performed, and we also analyzed ectized blood and lymphatic vessels. Muscle fibers have optically empty intracytoplasmic vacuoles (glycogen accumulations) of variable sizes. No pathological changes were detected.

We should mention that the existence of glycogenosis in the liver and heart was also observed in the control groups that did not receive implants, so it is not related to the implant materials.

Histopathological examinations about bone and muscle are presented in Figure 10i,j, which show antero-lateral musculature sectioned longitudinally parallel to the axis of the humerus. After the decalcification of the bone (24 h), three slices parallel to the longitudinal axis were made, of which the one that included the trajectory of the implant was included.

Focal periosteal reaction with proliferation of fibroblasts and osteogenic layer, abundant adjacent fibrous tissue with reduced cellularity and a reduced number of capillaries of uniform diameter, perpendicular or oblique to the surface of the periosteum (scar tissue). The bone compact in the vicinity of the implant shows an irregular subperiosteal surface, well-represented osteoid tissue, with rare, reduced foci of chondrocytes. Growth cartilage, scapulohumeral articular surface and bone marrow do not show changes.

The muscles show discrete lipid infiltration and numerous eosinophilic cells focally and peripherally aggregated among the connective tissue fibers, around a group of foamy macrophages. Eosinophils are also present perivascularly, in variable numbers, and multifocal.

Interpretation: Periosteal hyperplasia. Callus in resolution. Discreet eosinophilic myositis. Osseointegration is good, without inflammatory reactions or other adverse effects. The presence of eosinophils in the tissue may indicate a parasitic infection or a hypersensitivity response.

## 3. Materials and Methods

### 3.1. Fabrication of the Structure for Implantation

**Figure 11:**
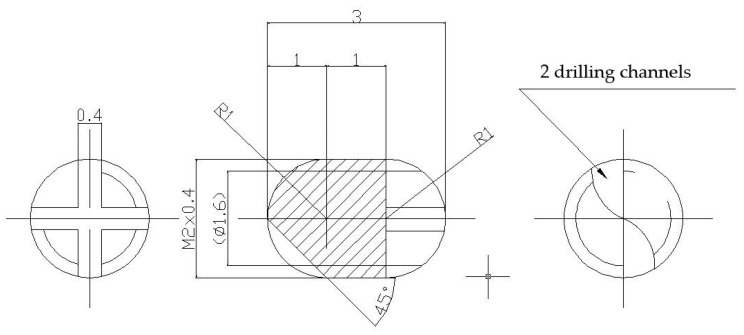
Metallic screws for implantation.

Were made two lots of metallic screws—one lot of stainless-steel screws and one lot of titanium grade 4 screws—with the proper dimensions for implantation: 3 mm long and 2 mm diameter, with M2 thread and a rounded tip achieved with 2 drilling channels (Figure 11). The implants were screwed with a cross key specially made for this experiment. The stainless-steel screws composition is as follows: 64.26% Fe, 18.51% Cr, 13% Ni, 2.13% Mo, 1.44% Mn, 0.48% Cu, 0.56% Si, 0.0265% C, 0.0036% S, and a smaller concentration of other elements, in accordance with the ISO 5832-1:2016(E) International Standard [56].

Some of the implants were covered with nanometric films containing bioactive glass (BG) from SiO_2_-Na_2_O-K_2_O-CaO-MgO-P_2_O_5_ system, plant extract of Azadirachta indica (neem), an Indian medicinal plant used in traditional Ayurveda treatment of various infections, and Poly(methyl methacrylate) (PMMA) in two layered structures- BG + neem/PMMA/OL- using an advanced laser-based technique (matrix assisted pulsed laser evaporation), like in [18].

### 3.2. Surgery and Implantation Procedure

**Figure 12:**
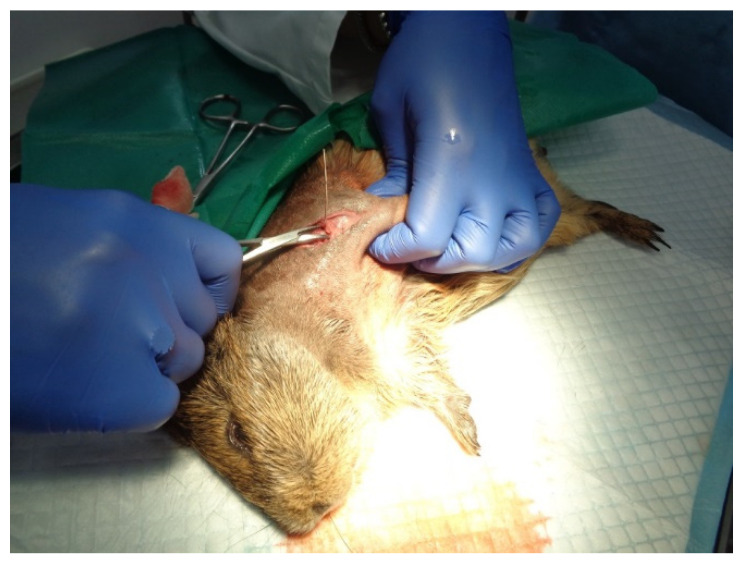
Implant surgery.

For our in vivo studies, the chosen subjects for implant surgery (Figure 12) were guinea pigs (*Cavia porcellus*), which have similar developmental and physiological characteristics to humans and, in some ways, are better models than mice because of their relatively larger body size and higher stress resistance to experimental manipulation [57]. The studies were carried out at the Department of Sanitary, Veterinary and Public Health of Brasov County (DSVSA). 

From the existing biobase, healthy animals (males) of the same age (one year) and approximately the same weight (650–700 g) were selected and grown in optimal conditions, as required by Law 149/2019 on the protection of animals used for scientific purposes, published in Official Monitor, MO, Part I nr. 619 of 25 June 2019, and also EU regulations on animal research [24,57,58]. Ten guinea pigs for each material and ten control guinea pigs were used.

Special cages with a minimum enclosure area of 2500 cm^2^, a minimum enclosure height of 23 cm, and a floor area of 900 cm^2^ per animal were constructed. The heating, ventilation, and lighting systems were inspected to ensure that the inside of the cages had a constant temperature of 22 °C, constant humidity, and 12 h light/12 h darkness cycles.

Important notes:

- The persons conducting or participating in the experiments, as well as the persons who cared for the animals, including those that supervised them, had received specialized training and had experience with such studies.
- A minimum number of animals of low neurophysiological sensitivity (guinea pigs) was used.
- All experiments were set up to prevent the experimental animals from suffering and experiencing undue pain; hence, general anesthesia was used, and if the animals suffered pain after the effect of anesthesia had passed, they were treated early with analgesics applied for 5 days post-operation.

### 3.3. In Vivo Study 

Screws consisting of the tested materials (steel, titanium, and steel coated with special thin films) were implanted into the humerus bones of guinea pigs from three groups, and the fourth group contained animals without implants, as shown in Table 4.

In the beginning, the pigs were weighed before being placed into a tucked position to enable one to chip into the implantation area. Kanamycin sulfate ophthalmic ointment (Antibiotice a+, Iasi, Romania) was applied to prevent corneal dryness during the surgery. Implantation was performed under general anesthesia by the neuroleptic analgesic method using a combination of ketamine (80 mg/kg body weight) and xylazine (10 mg/kg body weight), following ISO 10993-6:2016 [24].

Before the effect of anesthesia had finished and for 5 days after the surgery, analgesic injections (meloxicam) were applied for pain relief, and local treatment with both povidone iodine and spray containing zinc was applied to avoid infections.

At the end of the intervention, each guinea pig was microcipated for full control and handling without the risk of mistaking their belonging to one of the lots. X-ray post-implantation radiographs were made to visualize the correct insertion of the implant into the body.

For 26 weeks, the animals were kept, cared for, and supervised in accordance with the Care and Housing Standards set out in ANNEX NO. 3, Law 149/2019 [57].

Remarks:

- The wounds of guinea pigs from Lot III healed after 7 days;
- After 7 days, 2 guinea pigs from Lot I required sewing to close their wounds and anti-inflammatory treatment because they had a 1–2 cm long sore wound;
- One guinea pig from Lot II showed a 2 cm wound with a slight amount of blood-swelling exudate and needed 2-wire emergency stitching, as well as an anti-inflammatory injection;
- The occurrence of wounds may have been due to the stitching initially carried out on the inside, which proved to be too weak. It may be useful in subsequent studies to apply a “stitch in points” approach, which can lead to greater resistance.

To draw conclusions related to the influence of the implant on the guinea pigs’ body, X-ray and energy-dispersive spectroscopy (EDS) analyses were performed, as well as blood analyses, five months after implantation.

EDS was performed on all samples removed from the body after 26 weeks using a SiLi detector (EDAX Inc., Philips, Edinburgh, The Netherlands) that has eZAF Smart Quant Results software and operates at 15 kV. 

Some blood was used immediately after harvesting to quantify urea, creatinine, glycemia, alkaline phosphatase, pancreatic amylase, total serum protein, and glutamate pyruvate transaminase to analyze how the body was affected by implant insertion. 

### 3.4. Detection of Enzymatic Activity

Moreover, another part of the collected blood was harvested in EDTA anticoagulant containers and centrifuged at 1000× *g* for 10 min to obtain plasma, which was then stored at −20 °C. Using special kits (Cayman Chemical, Ann Arbor, Mi, USA), enzyme activity was evaluated by measuring superoxide dismutase, catalase, and glutathione peroxidase. Changes in the values of these parameters compared to the non-implanted group will indicate the level of oxidative stress in the body and the degree of damage to the body caused by inserting the implant, and comparisons with the group of guinea pigs implanted with SS will show whether there are any advantages of implant coating.

The introduction of an orthopedic implant into the body triggers an inflammatory response as a response of the immune system to the presence of a foreign body. This inflammatory response involves the release of pro-inflammatory cytokines such as interleukin-1 (IL-1), interleukin-6 (IL-6), and tumor necrosis factor-alpha (TNF-α), as well as the formation of reactive oxygen species (ROS). These reactions can impact the performance and durability of orthopedic implants.

Superoxide dismutase (SOD), glutathione peroxidase (GPx), and catalase (CAT) are antioxidant enzymes involved in neutralizing and eliminating reactive oxygen species from the body. These enzymes have an important role in protecting tissues against oxidative stress and may be relevant in the context of coated implants.

Their activity is often measured to assess the level of oxidative stress induced by orthopedic implants. Oxidative stress occurs when there is an imbalance between the production of reactive oxygen species and the ability of the body’s antioxidant system to neutralize them. Enzyme activity is therefore considered an important marker of the cellular ability to combat superoxide free radicals and maintain the redox balance around the implants.

#### 3.4.1. Determination of Superoxide Dismutase

In this work, tetrazolium salt was utilized for the detection of superoxide radicals in blood plasma. One unit of SOD is defined as the amount of enzyme needed to exhibit 50% dismutation of the superoxide radical. Triplicate standards and samples were put on a well plate up to a volume of 210 μL in a well-determined order.

Into the SOD standard wells, 10 μL of the standard solution of bovine erythrocyte (Cu/Zn) with 7 different concentrations (and activity) and 200 μL of the diluted Radical Detector (tetrazolium salt solution) were added. The sample wells contained 10 μL of sample and 200 μL of the diluted Radical Detector. A reaction was initiated by adding 20 μL of diluted xanthine oxidase to all the wells; the well plates were shaken for a few seconds and incubated on a shaker at room temperature for 30 min. Then, the absorbance was read at 440–460 nm using a plate reader.

**Figure 13:**
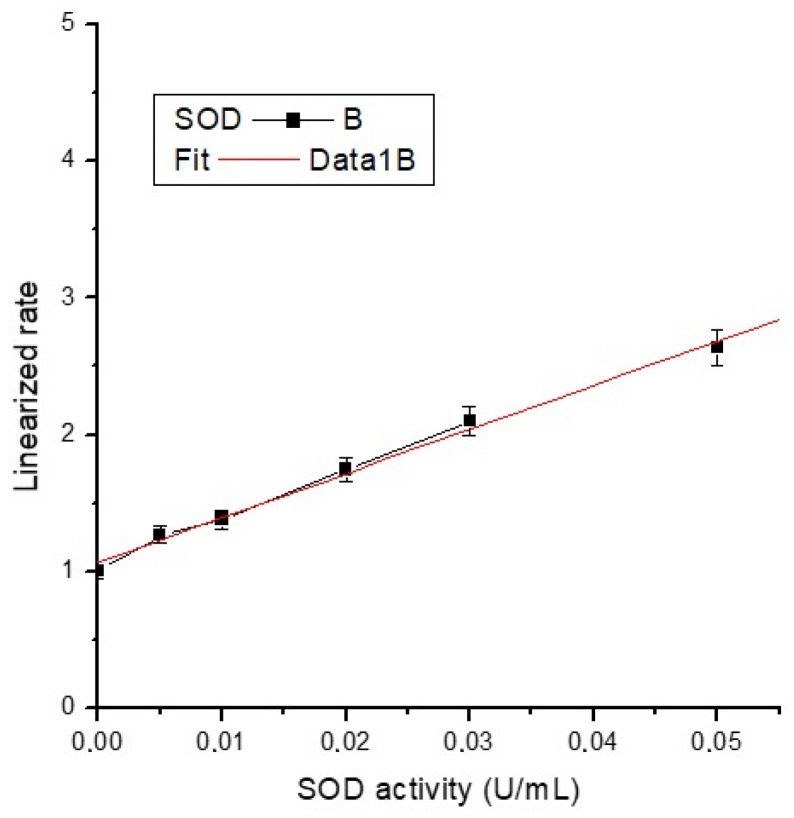
Calibration curve of SOD activity.

The average absorbance of each standard and sample was calculated, and sample background absorbance was subtracted from the sample. The activity of superoxide dismutase was determined (Table 3) using the formula below (where the necessary corrections are made) using the slope of the calibration curve (Figure 13) and the linearized SOD standard rate (LR) as a function of final SOD Activity (U/mL):

$$SOD\left(\frac{U}{mL}\right)=\left[\left(\frac{sample\;LR-y\;intercept}{slope}\right)\times \frac{0.23\;mL}{0.01\;mL}\right]\times sample\;dilution$$

The equation of the calibration line is y = 1.06582 + 32.38154x, with a very good correlation coefficient (R^2^ = 0.997), almost unitary.

#### 3.4.2. Determination of Catalase

We used the peroxidatic function of CAT for the determination of enzyme activity. This method is based on the reaction between the enzyme and methanol in the presence of an optimal H_2_O_2_ concentration. The formaldehyde produced is measured using 4-amino-3-hydrazino-5-mercapto-1,2,4-triazole (Purpald) as the chromogen. 

The preparation of the formaldehyde standards was carried out by diluting catalase formaldehyde standard with diluted sample buffer to obtain formaldehyde stock solution with different concentrations: 0, 5, 15, 30, 45, 60, and 75 µM formaldehyde.

The standards, positive control (lyophilized powder of bovine liver CAT), and samples were put in triplicate on a well plate up to a volume of 240 μL in a very well-established order, following the assay protocol. The reactions were initiated by adding 20 μL of diluted hydrogen peroxide to all the wells, and after 20 min of incubation at room temperature, 30 μL potassium hydroxide was added to each well to end the reactions. We put 30 μL of catalase Purpald (chromogen) in each well, followed by incubation on a shaker for 10 min at room temperature. 

Absorbance was read at 540 nm, and the calibration curve was built. Using the curve slope and the formula below, with necessary corrections, formaldehyde was determined.

$$Formaldechyde\left(\mu M\right)=\left[\frac{sample\;absorbance-y\;intercept}{slope}\right]\times \frac{0.17\;mL}{0.02\;mL}$$

The CAT activity of the sample was calculated (Table 3) using the following equation and considering that one unit is the amount of enzyme that will cause the formation of 1.0 nmol of formaldehyde per minute at 25 °C.

$$CAT\;activity=\frac{µm\;of\;Formaldehide\;sample}{20\;min}\times sample\;dilution=\frac{nmoL}{min}/mL$$

#### 3.4.3. Determination of Glutathione Peroxidase

In this work, GPX activity was measured indirectly by the oxidation of NADPH, which was accompanied by a decrease in absorbance at 340 nm.

On a well plate, in triplicate, the background (assay buffer), positive controls (bovine erythrocyte GPX, lyophilized glutathione, and glutathione reductase), and the samples were measured up to a volume of 190 μL in a very well- determined order, and the reaction was initiated by quickly adding 20 µL of cumene hydroperoxide to all the wells.

Absorbance was read at 340 nm once per minute to obtain more than five readings; the mean absorbance was calculated, and the calibration curve for each reading was constructed. Using its slope and the formula below, where the necessary corrections are made, GPX activity was determined (Table 3).

$$GPx\;activity=\frac{slope}{0.00373}\times \frac{0.19\;mL}{0.02\;mL}\times sample\;dilution$$

### 3.5. Histopathological Evaluation 

The histopathological evaluation of orthopedic medical devices involves the examination of tissue samples collected from around the implant site to assess the local tissue response and potential adverse reactions associated with the device, as well as the examination of vital organs, including the heart, liver, pancreas, spleen, and kidneys. This comprehensive evaluation provides a broader understanding of the systemic effects and potential adverse reactions associated with the medical device [59,60,61]. It is an important component of preclinical and clinical studies to evaluate the biocompatibility and safety of orthopedic implants. 

Our histopathological evaluation of the different types of studied implants had multiple aims, the first of which was to assess tissue response to the implant. This involved examining the cellular and structural changes, inflammation, fibrosis, vascularization, and presence of foreign body reactions in the tissue surrounding the implant. This evaluation provided insights into the biocompatibility and local tissue integration of the device [62].

The second aim was the identification of adverse reactions. Histopathological evaluations help identify any adverse reactions or complications associated with the orthopedic device. This may include the presence of necrosis, granuloma formation, chronic inflammation, excessive fibrosis, or other tissue abnormalities. The detection of adverse reactions is crucial for understanding the potential risks and improving the design and performance of medical devices and is of great importance in determining the possible hospital-associated infections [63].

The third goal was the assessment of the potential systemic effects and toxicity caused in vital organs, which involves any pathological changes, inflammation, or organ-specific reactions that may result from the presence of the implant, as well as organ-specific toxicity such as hepatotoxicity, nephrotoxicity, and cardiotoxicity, which may occur due to the implant or its interaction with the body. Our histopathological evaluation of vital organs also provided information on organ function and viability. It helped to assess the impact of the implant on organ health and functioning, allowing for the detection of any abnormalities or functional impairments.

The procedure used for our histopathological evaluation was the usual one. Tissue samples were collected from the implant site during post-mortem examination. The samples included samples of peri-implant tissue, adjacent bone, surrounding soft tissue, and vital organs. The collected tissue samples were carefully dissected, fixed, processed, and embedded in paraffin wax or frozen for sectioning. Paraffin sections were stained with histological stains like Hematoxylin and Eosin (H&E) for general tissue assessment, while other specialized stains were used to evaluate specific tissue components or reactions. Then, the stained tissue sections were examined under a microscope.

## 4. Conclusions

Our biochemical blood analysis and histological analysis of the guinea pigs’ organs and of the tissues surrounding the implants did not show significant adverse effects caused by the implant coating containing bioactive glass, a polymer, and natural plant extract in terms of tissue compatibility, inflammatory reactions, and systemic effects.

## Figures and Tables

**Table 1:** Chemical compositions in wt% of the coatings obtained by MAPLE.

Sample	Element									
	Ca	P	Na	Mg	C	O	Al	Cr	Fe	Ni
	Concentration (wt. %)									
BGN	8.56	6.58	1.9	2.82	16.83	41.7	0.28	3.37	11.59	1.01

**Table 2:** Blood analysis results for study groups.

Analysis	Normal Values	Lot I Ti	Lot II OL	Lot III BGN	Lot IV WI
Urea (mmol/L)	2.04–11.28	3.21	3.07	3.13	3.13
Creatinine (µmol/L)	23.90–73.45	59.4	58.67	52.8	61.6
Blood glucose (mmol/L)	4.62–19.55	15.44	12.90	14.74	10.22
Pancreatic amylase (U/L)	726.93–1831.55	1421	1420.33	1479	1707.25
Total protein (g/dL)	5.00–7.09	4.85	4.27	4.25	4.88
Alkaline phosphatase (U/L)	50.80–328.10	42	28	27	41
TGP (U/L)	41.45–165.35	23	22.33	21	22.25

**Table 3:** Calculated values for enzymatic activity.

Lot	SOD (U/mL)	CAT (nmol/min/mL)	GPx (nmol/min/mL)
I Ti	0.244	13.98	0.480
II OL	0.250	17.26	0.462
III BGN	0.270	18.39	0.486
IV WI	0.206	14.43	0.739

**Table 4:** The study groups.

Group	Substrate	Coating	Name
I	Ti	without coating	Ti
II	OL	without coating	OL
III	OL	BG + neem/PMMA	BGN
IV	unimplanted guineea pigs		WI

## Data Availability

The raw data supporting the conclusions of this article will be made available by the authors on request.
